# Navigating complexity: a comprehensive review of microcatheter shaping techniques in endovascular aneurysm embolization

**DOI:** 10.3389/fneur.2023.1245817

**Published:** 2023-10-19

**Authors:** Changya Liu, Xinxin Wu, Xuebin Hu, Linguangjin Wu, Kaikai Guo, Shuang Zhou, Bangjiang Fang

**Affiliations:** ^1^Department of Emergency, Longhua Hospital, Shanghai University of Traditional Chinese Medicine, Shanghai, China; ^2^Shanghai Skin Disease Hospital, Skin Disease Hospital of Tongji University, Shanghai, China; ^3^Department of Neurosurgery, Union Hospital, Tongji Medical College, Huazhong University of Science and Technology, Wuhan, Hubei, China; ^4^School of Acupuncture-Moxibustion and Tuina, Shanghai University of Traditional Chinese Medicine, Shanghai, China; ^5^Institute of Critical Care, Shanghai University of Traditional Chinese Medicine, Shanghai, China

**Keywords:** endovascular, intracranial aneurysm, microcatheter shaping, techniques, embolization

## Abstract

The endovascular intervention technique has gained prominence in the treatment of intracranial aneurysms due to its minimal invasiveness and shorter recovery time. A critical step of the intervention is the shaping of the microcatheter, which ensures its accurate placement and stability within the aneurysm sac. This is vital for enhancing coil placement and minimizing the risk of catheter kickback during the coiling process. Currently, microcatheter shaping is primarily reliant on the operator's experience, who shapes them based on the curvature of the target vessel and aneurysm location, utilizing 3D rotational angiography or CT angiography. Some researchers have documented their experiences with conventional shaping methods. Additionally, some scholars have explored auxiliary techniques such as 3D printing and computer simulations to facilitate microcatheter shaping. However, the shaping of microcatheters can still pose challenges, especially in cases with complex anatomical structures or very small aneurysms, and even experienced operators may encounter difficulties, and there has been a lack of a holistic summary of microcatheter shaping techniques in the literature. In this article, we present a review of the literature from 1994 to 2023 on microcatheter shaping techniques in endovascular aneurysm embolization. Our review aims to present a thorough overview of the various experiences and techniques shared by researchers over the last 3 decades, provides an analysis of shaping methods, and serves as an invaluable resource for both novice and experienced practitioners, highlighting the significance of understanding and mastering this technique for successful endovascular intervention in intracranial aneurysms.

## Introduction

In light of recent advancements in technology and material sciences, endovascular intervention techniques have emerged as significant therapeutic modalities for managing intracranial aneurysms (1). These techniques have garnered preference from both patients and clinicians owing to their minimally invasive nature and expedited recovery period (2, 3). Nonetheless, the execution of endovascular embolization therapy, whether it employs simple coil embolization or is supplemented by stents or balloons, faces a principal technical challenge in accurately navigating the microcatheter into the aneurysm sac and ensuring its stability therein (4). Optimal shaping of the microcatheter enhances its stability within the aneurysm sac, promotes effective coil placement, and curtails the risk of catheter kickback during the coiling process. Therefore, in order to successfully complete intracranial aneurysm embolization, shaping the microcatheter into an appropriate form is crucial, and achieving proper microcatheter shaping is a critical step (5, 6).

At present, the procedure of microcatheter shaping primarily depends on the experience of the operator, who bends them to approximate the desired shape according to the curvature of the target vessel and the position of the aneurysm with three-dimensional (3D) rotational angiography or computed tomography angiography (CTA) (7). Notably, in exceptional instances involving complex anatomical structures or diminutive aneurysms, even experienced operators might have to reshape the microcatheter multiple times or face challenges in accomplishing successful shaping (8). Additionally, in the process of evolving from an inexperienced novice to a skilled interventionalist, understanding comprehensively the technique of microcatheter shaping, and gradually accumulating experience, is also an important step in learning the technique of endovascular intervention (9). Hence, despite microcatheter shaping being a routine step in endovascular aneurysm embolization, its implementation can be challenging.

Concerning the topic of microcatheter shaping, diverse researchers have contributed their experiences with conventional shaping methods. Moreover, certain scholars have utilized auxiliary techniques such as 3D printing and computer simulations to assist with microcatheter shaping (10, 11). Despite these contributions, a comprehensive synthesis of microcatheter shaping techniques, to the best of our knowledge, is still lacking in scholarly literature. Consequently, this article aims to collate and review previously reported techniques in microcatheter shaping.

## Methods and search strategy

**Figure 1:**
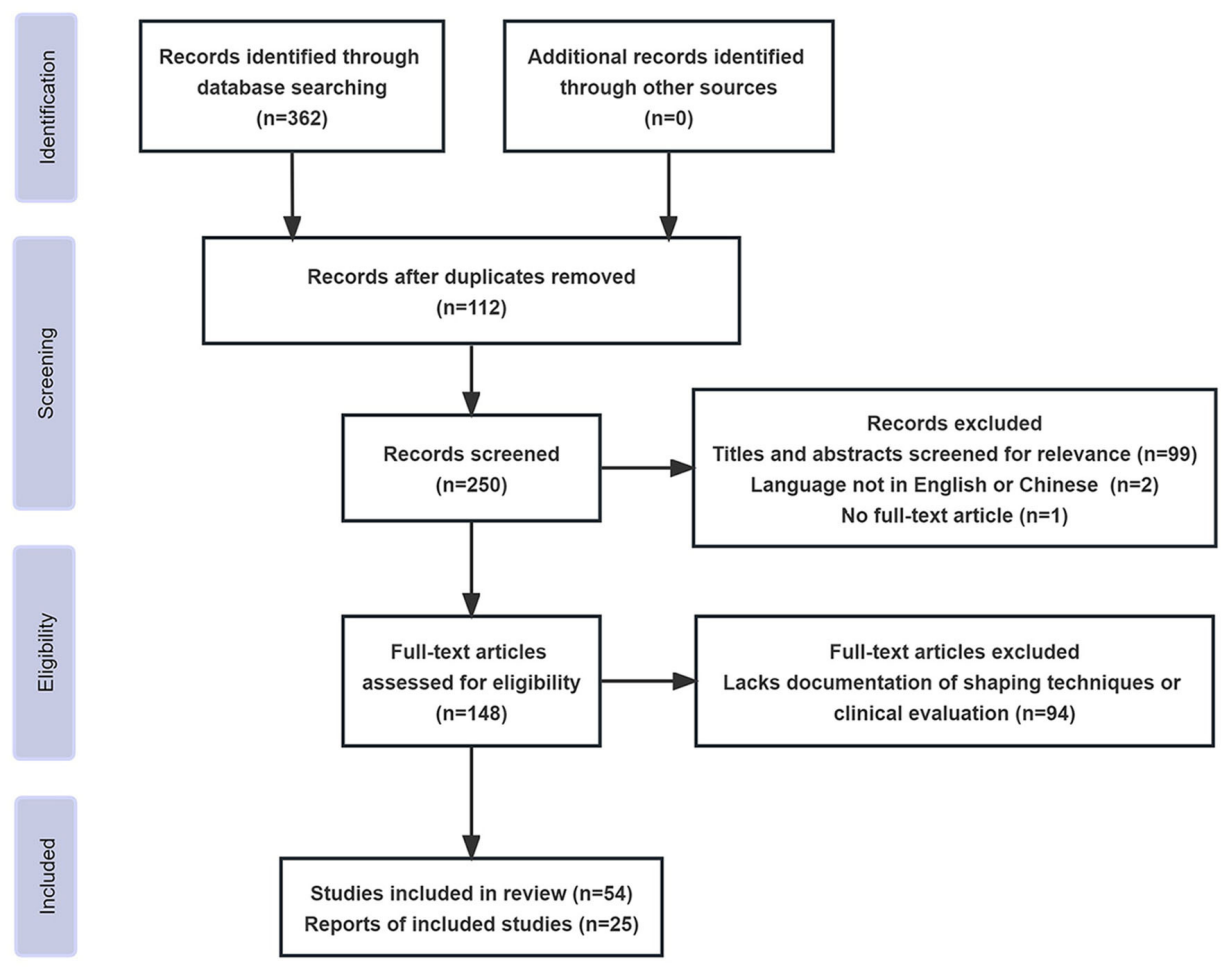
Flow diagram for literature screening.

The literature search was performed to identify studies regarding the application of microcatheter shaping in intracranial aneurysm embolization. We identified key terms and phrases related to microcatheter shaping in the treatment of intracranial aneurysms, such as “Microcatheter,” “Shaping,” “Intracranial Aneurysms,” “Endovascular Treatment,” and “Neurointervention.” We combined these terms using Boolean operators to formulate our search strings. The literature search was conducted based on the PubMed, Web of Science, Scopus, ScienceDirect, J-STAGE, and CNKI databases for relevant publications from January 1994 to May 2023. Additionally, we utilized Google Scholar to ensure broader coverage. Literature types included all English and Chinese articles, such as original articles, case reports, and reviews. Meta-analyses were not included as there were no published ones in this field. After completing the data retrieval, two authors independently screened the titles and abstracts of the literature, while all authors collectively assessed the full texts of potentially relevant articles and whether they could be included in our study (illustrated in Figure 1).

### The shapability of different microcatheters

Initiating the microcatheter shaping process necessitates an in-depth comprehension of the shapability performance of different microcatheter types. In the past, several publications have reported scholarly research focusing on this particular aspect (Table 1). The first literature report on the comparative study of shapability performance of different types of microcatheters was illustrated by Abe et al. (12). In their study, the distal tip of five types of microcatheters were shaped into a 90° turn with distal straight segment lengths of 3, 5, or 7 mm, respectively. The authors investigated the modifications in the curvature of the shaped microcatheters under various conditions: post-insertion into a guiding catheter, post-microguidewire insertion through the microcatheter, and post-Guglielmi detachable coil (GDC) placement through the microcatheter. Kiyosue et al. (13) conducted an evaluation and comparison of 12 microcatheter types. Following the insertion of shaping mandrels into the microcatheters, the distal tips were flexed at 90° and 150°, respectively, and measured using a goniometer. The microcatheters were then exposed to steam for 20 s, followed by immersion in 17°C water for an additional 20 s. Upon the removal of the shaping mandrels, the shaping angles were measured and subjected to further analysis.

**Table 1:** Summary of published studies on the shapability performance of microcatheters.

**Study**	**Type of microcatheters**	**Shaping angles**	**Bending length**	**Steam method**	**Stability test method**
Abe et al. (14)	FasTracker-10, Excel-14, Excelsior SL-10, Prowler-14	90°	5 mm	Steaming then placed in water	Inserting microguidewires 10 times
Abe et al. (12)	FasTracker-10, Excel-14, Excelsior SL-10, Prowler-14, Prowler-14 Preshaped	90° 90°	3 mm,	Steaming then placed in 37°C water	Inserting microguidewires 10 times,
Kiyosue et al. (13)	Excelsior 1018, Tracker Excel-14, Excelsior SL-10, Progreat 2.0F, Rebar-14, FasTracker-10, Rapid Transit, Prowler Plus, Renegade-18, Progreat 2.4F, Microferret, Prowler Plus MX	90°,	5 mm	Steaming for 20s and placed in 17°C water for 20 seconds	Inserting microguidewires 5 times
Fujimoto et al. (15)	Excelsior SL-10, Echelon-10, Headway-17, Excelsior XT-17	180°, 360°, 720°	4 mm (diameter)	Steaming for 30 s and placed in 37°C physiological saline	Inserting microguidewires 10 times
Wattanasatesiri et al. (16)	Progreat lambda 1.7F, Progreat alpha 2.0F, Veloute 1.7F, Radiostar 1.9F, Carnelian 1.8F	90° (L shape), 180° (U shape), 360° (O shape)	5 mm	Steaming (80°C) for 60 s and then placed in 24°C water for 20 s	Passing through a 5F catheter and inserting microguidewires

GDC, Guglielmi detachable coil.

**Figure 2:**
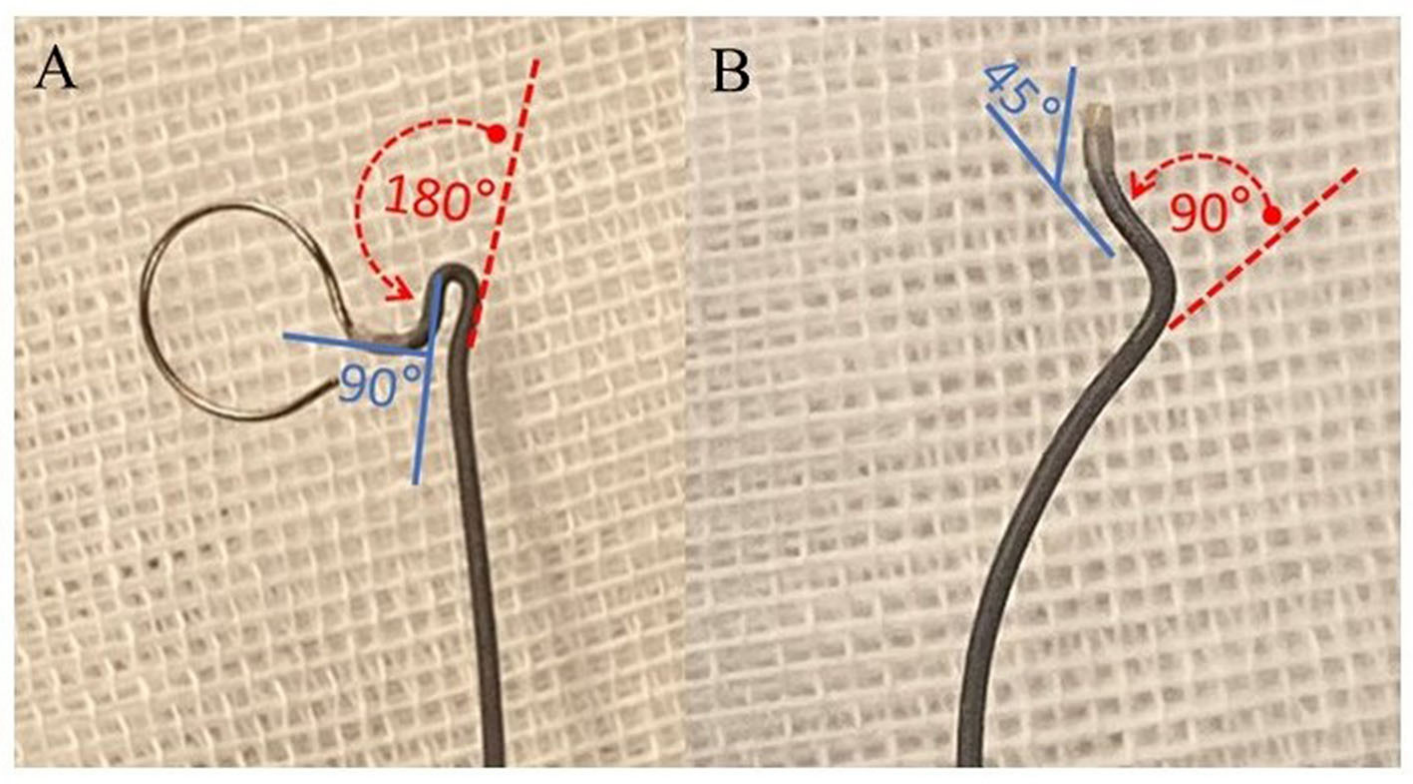
Diagram illustrating the shaping ratio of the microcatheter. **(A)** Insert the mandrel into a linear Echelon microcatheter. Subsequently, flex the segment proximal to the tip to an angle of 90° (indicated by the blue solid line) and the segment distal to the tip to 180° (denoted by the red dashed arrow). **(B)** Upon co-heating the microcatheter with the mandrel for shaping and subsequently extracting the mandrel, the segment of the microcatheter proximate to the tip rebounds to an approximate angle of 45° (blue solid line), while the segment distal to the tip rebounds to an angle of 90° (red dashed arrow). This attribute is denominated as the capability to achieve a 1:2 shaping ratio.

In another study, Fujimoto et al. (15) assessed the shapability of four microcatheter types, including Excelsior SL-10 (Stryker, Kalamazoo, MI, USA), Echelon-10 (Medtronic, Minneapolis, MN, USA), XT-17 (Stryker, Kalamazoo, MI, USA), and Headway-17 (MicroVention TERUMO, Tustin, CA, USA). In their method, a mandrel was inserted into a microcatheter, and the distal end was coiled with a diameter of 4 mm for 1, 2, and 3 turns, respectively, before being steam-shaped for 30 s. The microcatheter was subsequently immersed in 37°C physiological saline for 10 min. Subsequently, a microguidewire was inserted into the microcatheter, extending 2 cm from the distal end of the microcatheter. Their findings demonstrated that immediately after steam shaping and removal of the mandrel, all four types of microcatheters, when coiled for one turn, displayed a semi-circular shape (204°, average diameter of 8.7 mm). Those coiled for two turns had an average remaining coil of 1.2 turns (472°, average diameter of 7.7 mm), while the ones coiled for three turns had an average remaining coil of 1.8 turns (671°, average diameter of 7.9 mm). No significant differences in morphology were observed among the three shaping conditions. Nevertheless, after immersion in physiological saline for 10 min, there were substantial morphological changes in Excelsior SL-10 and Echelon-10 microcatheters, whereas Headway-17 and XT-17 microcatheters exhibited better shape retention capabilities. Despite the numerous types of microcatheters available in the current market, research on the performance of microcatheters remains limited, and the understanding of shaping techniques is largely dependent on the personal experience of interventionalists. Based on our center's experience, we believe that the Headway-17 microcatheter can achieve a 1:1 shaping ratio based on the angle between the parent artery and the long axis of the aneurysm. Additionally, microcatheters such as Excelsior SL-10, Echelon-10, and Echelon-14 can achieve a 1:2 shaping ratio (illustrated in Figure 2).

### The heating condition for shaping

After manipulating the mandrel, the usual procedure for interventionalists involves applying heat for a certain duration, then immersing it in cold water, with the expectation that the distal end of the microcatheter will maintain this particular shape reliably. Akihiko et al. (17) explored this issue of heating methods in their research. They examined the effects of different heating durations on the shaping of the headway microcatheter using four commonly used equipment, namely, a heating plate + kettle, an electric kettle, a steamer, and a hot air gun. The results indicated that under the temperature measurement condition at a distance of 25 mm, the heating plate + kettle, electric kettle, and steamer took a longer time to reach a stable peak temperature, which were 80, 95, and 60°C, respectively. In contrast, the hot air gun reached a stable peak temperature rapidly upon activation though the actual measured temperature was 20°C below the set value. Regarding the effect of heating on the microcatheter, their result indicated that the headway microcatheter retained its optimal shaping after being subjected to heating at 100°C for 90 s, with no significant surface damage observed. Hence, they concluded that using a hot air gun to maintain a heating duration of 90 s at 100°C resulted in the best shaping effect for the microcatheter. In another study, Tomio et al. (18) explored the optimal shaping conditions for Echelon-10 and Excelsior SL-10 microcatheters using a similar methodology. Their result revealed that by subjecting the microcatheters to continuous heating for 30 s at a set temperature of 120°C using a hot air gun, both types of microcatheters achieved the desired shaping curvature under a twice-bending status. Additionally, these microcatheters were able to endure 2–3 reshaping cycles under this heating condition.

### Conventional microcatheter shaping method

In the current process of endovascular embolization for intracranial aneurysms, the prevalent method of microcatheter shaping involves the operator, utilizing three-dimensional reconstructed images after completion digital subtraction angiography (DSA) examination, in conjunction with their own experience, to manually shape the microcatheter by inserting a mandrel into the tip of the microcatheter and manipulating it to match the morphological traits of the lesion site. Over the past few decades, some scholars have analyzed and reported on these methods and experiences (Table 2). Kwon et al. (19) shared their experience of microcatheter shaping in the treatment of paraclinoid aneurysms. They encompassed 132 paraclinoid aneurysms and categorized the shaping forms into straight, primary curves (45°, 90°, and J-shaped), C-shaped, pigtail-shaped, and S-shaped. For paraclinoid aneurysms growing superiorly or medially, they found the S-shaped (54.5%) and pigtail-shaped (60.2%) forms to be the most frequently used, respectively. After analysis, they suggested that an S-shaped for superiorly directed aneurysms and a pigtail-shaped for medially directed aneurysms appear to be suitable for microcatheter shapes. In a further delineation, Wang et al. (20) subdivided paraclinoid aneurysms into four subtypes based on location and direction and proposed four corresponding microcatheter shaping strategies: “straight shaped,” “S-shaped,” “C-shaped,” and “primary curve.”

**Table 2:** Summary of published studies on the conventional shaping method.

**Study**	**Type of articles**	**Number of aneurysms**	**Location**	**Shaping strategy**	**Type of microcatheters**	**Result measurement**
Toyota et al. (21)	Cohort	10	Paraclinoid	3D rotational angiography and volume-rendering images on the monitor	Excelsior SL-10	-
Kwon et al. (19)	Cohort	132	Paraclinoid	Straight 6 (4.5%)	Excelsior 1018, Excelsior SL-10, Prowler-14, Prowler Select	Complete occlusion 76 (57.6%)
				Primary shape (45°/90°/J) 34 (25.8%)		Neck remnant 43 (32.6%)
				C-preshaped 13 (9.8%)		Residual aneurysm 13 (9.8%)
				Pigtail-shaped 58 (43.9%)		
				S-shaped 21 (15.9%)		
Chang et al. (22)	Cohort	13	A1	Tailored shape,	-	Complete occlusion 8 (2.7%)
				S-shaped		Neck remnant 2 (15.4%)
						Residual aneurysm 3 (23.1%)
Lee et al. (23)	Case report	4	A1	Z-shaped 2 (50.0%)	Excelsior SL-10	Complete occlusion 2 (50.0%)
				S-shaped 2 (50.0%)		Residual aneurysm 1 (25.0%)
						Abort operation 1 (25.0%)
Xiaochuan et al. (24)	Cohort	15	A1	S-shaped 7 (46.7%)	Echelon-10, Headway-17	Complete occlusion 15 (100%)
				Z-shaped 2 (13.3%)		
				U-shaped 2 (13.3%)		
				No shaped 4 (26.7%)		
Wang et al. (20)	Cohort	64	Straight	Echelon-10, Echelon-14, Excelsior SL-10, Prowler-14	Complete occlusion 23 (35.9%)
				S-shaped	Neck remnant 22 (34.4%)
				C-shaped	Residual aneurysm 19 (29.7%)
				Primary shape	
Ko et al. (25)	Cohort	11	A1	S-shaped	-	Complete occlusion in 11 (100%)
				Z-shaped		
Cho et al. (26)	Cohort	50	A1	Straight 10 (20%)	Excelsior SL-10	Complete occlusion/Neck remnant 38 (76.0%)
				S-shaped 30 (60%)		Residual aneurysm 12 (24.0%)
				Primary shape (45/90°) 10 (20%)		
Ahn et al. (27)	Cohort	43	OA	Straight 5 (11.6%)	-	Complete occlusion 14 (32.6%)
				S-shaped 21 (48.8%)		Neck remnant 23 (53.5%)
				Pigtail-shaped 14 (32.6%)		Residual aneurysm 6 (13.9%)
				Primary shape (45°/90°) 3 (6.9%)		
Cho et al. (28)	Cohort	59	Paraclinoid	Pigtail-shaped 41 (69.5%)	Excelsior SL-10	Complete occlusion 9 (15.3%)
				Primary shape (45°/90°) 11 (18.6%)		Neck remnant 35 (59.3%)
				S-shaped 7 (11.9%)		Residual aneurysm 15 (25.4%)
Shiwei et al. (29)	Cohort	51	PcomA	3D rotational angiography and volume-rendering images on the monitor	-	Complete occlusion 43 (84.3%)
Neck remnant 8 (15.7%)
Chung et al. (30)	Cohort	80	Paraclinoid	S-shaped	Excelsior SL-10	-
				J-shaped		
				C-shaped		

A1, the A1 segment of the anterior cerebral artery; OA, the ophthalmic artery segment of the internal carotid artery; PcomA, posterior communicating artery.

For proximal anterior cerebral artery aneurysms, Chang et al. (22) underlined the necessity of tailored-shaped or S-shaped microcatheters based on three-dimensional reconstructed images. In another study on endovascular treatment of proximal anterior cerebral artery aneurysms, Lee et al. (23) utilized various microcatheter shaping forms and deduced that a Z-shaped morphology at the tip of the microcatheter facilitated smooth access into the proximal aneurysmal sac in the A1 segment and provided a stable embolization pathway. In another study focused on 11 cases of anterior cerebral artery aneurysms, the authors also documented the application of “S-shaped” or “Z-shaped” microcatheter shaping strategies by the operators (25). Moreover, in a case series reported by Huo et al. (24) involved 15 patients with anterior cerebral artery aneurysms, the microcatheter-shaping strategies were further categorized into several types based on the location and growth direction of the aneurysms.

It is important to note that these conventional shaping methods largely originate from the accumulation of experiences and personal insights of different operators. Additionally, the cases reported in the above literature primarily focus on relatively common sites within the intracranial anterior circulation system. Therefore, the universal applicability of conventional shaping methods in addressing the individual characteristics of different cases, as well as complex cases, and the understanding and learning needs of inexperienced novices still pose considerable challenges.

### Improvement attempts on conventional shaping methods

Building upon the previously mentioned microcatheter shaping methods, some scholars have also attempted to make improvements in order to achieve greater stability of the microcatheter. Jia et al. (5) presented a microcatheter shaping technique called “loop technique” based on the principle of the interaction forces between the vessel wall opposite to the aneurysm neck and the curved portion of the proximal vessel wall with the microcatheter for coil embolization of paraclinoid aneurysms. In this technique, the distal part of the microcatheter was steam-shaped into a loop, forming the second curve, while the tip was further shaped to align with the long axis of the aneurysm, creating the first curve. The third curve was designed to align with the cavernous genu portion of the internal carotid artery (ICA). By utilizing the interaction forces, the aim was to enhance the stability of the microcatheter during the embolization procedure. However, the author also pointed out that this approach made it more challenging to adjust the microcatheter tip toward the aneurysmal sac. Additionally, this method carries risks of damaging the vessel wall and getting hooked with the coil loops.

Another improvement came from Ohshima et al. (31). Building upon the conventional shaping techniques, they introduced a modification where the distal 1–2 mm of the microcatheter tip was bent and formed a “Γ” shape. They observed that the microcatheters with the “Γ” tip demonstrated improved movement and oscillation during coiling and reduced coil protrusion into the parent artery and decreased microcatheter kickback. However, their study was limited to *in vitro* experiments and lacked corresponding clinical case applications. Additionally, based on our experience, the “Γ” tip carries the risk of getting hooked with coil loops, especially when coiling the neck of the aneurysm. If there was no prior plan for a stent-assisted or balloon-assisted approach, dealing with such an issue can be challenging once it arises.

Furthermore, Kwak et al. (32) reported a case of endovascular treatment for an ultrawide-necked circumferential aneurysm of the middle cerebral artery. During the procedure, the authors shaped two microcatheters into a spring form and positioned them at the distal portion of the aneurysm. The intention was for the microcatheters to coil around the stent after its deployment, allowing the coils to distribute evenly within the aneurysm sac. In this article, the authors proposed a novel microcatheter shaping technique for the treatment of circumferential aneurysms or fusiform aneurysms. However, it should be noted that the authors utilized the LVIS BLUE (MicroVention TERUMO, Tustin, CA, USA) stent, which was a flow-diverting device with a tight mesh design and minimized the coil encroachment into the stent. In this study, whether this method is applicable to laser-cut or braided stents was not addressed.

### Different attempts in the process of conventional shaping

As mentioned above, in the conventional microcatheter shaping process, the operator shaped and heat the microcatheter with inserting the mandrel into the tip. However, for complex lesion structures or cases requiring significant reshaping, the insertion of the mandrel may cause changes in the curvature of the microcatheter tip, leading to a reduction in shaping accuracy. Therefore, several scholars have attempted different approaches in the shaping process. Tomotaka et al. (33) proposed a shaping process named microcatheter shaping cast. In their report, the operators utilized a metallic introducer to coil the mandrel 4–5 times, creating a stent-like handmade cast. Subsequently, the microcatheter was inserted into the cast, and manual bending was performed according to the morphology of the lesion with a hot air gun for shaping. However, the authors of this article did not provide any demonstration of the practical application of this method in the actual case. Furthermore, we attempted to apply this method during endovascular treatment procedures but encountered difficulties in achieving uniform coiling of the mandrel and bending of the cast, which in turn affected the shaping process.

Another attempted process for forming the shape of the microcatheter was called “intravascular placement” or “endovascular shaping.” In Shinya et al. (34) reported on this method first in a series of 15 cases. They delivered an SL-10 straight microcatheter to the neck of the aneurysm and left it in the parent artery for 5 min. After pulling out the microcatheter, it acquired a certain curvature. Subsequently, based on the 3D reconstructed images of the vessel and aneurysm, the tip of the microcatheter was steam-shaped into the corresponding form. In another report, Katsunari et al. (35) used an XT-17 microcatheter and employed the same method to treat vertebral basilar aneurysms. Ultimately, accurate microcatheter shaping was achieved in all five cases, and the procedures were successfully completed. Moreover, Shinoda et al. (36) employed the same shaping method to treat a case of blister-like aneurysm. Yoshiki et al. (37) reviewed 10 cases of bifurcation aneurysms treated using this shaping method and similarly obtained satisfactory results. We believe that “endovascular shaping” can be attempted for cases with more complex structures or cases where multiple reshaping attempts have been unsatisfactory. However, compared to conventional shaping methods, an additional 5-min microcatheter delivery procedure theoretically increases the risk of complications such as vascular injury and intravascular thrombus formation. Furthermore, this procedure will prolong the duration of microcatheter shaping and overall operational time.

### Shaping based on 3D model reconstruction and printing technology

During the microcatheter shaping process, 3D rotational angiography provided assistance to the interventionalists in understanding the structural morphology of the lesion. However, this observation was achieved solely through a two-dimensional computer screen, lacking a correct depth perception (38). Therefore, some scholars have attempted to use *in vitro* 3D models and printing technology to gain a more comprehensive understanding and analysis of the lesion structure, thus aiding in the precise shaping of the microcatheter (Table 3). Kenichi et al. (39) reported for the first time the creation of a specific silicone vascular model of the lesion site in a patient with anterior communicating artery aneurysm based on 3D rotational angiography. They performed preoperative simulated treatment, focusing particularly on microcatheter navigation and tip shape. In another study, Katsunari et al. (40) reported the use of 3D printing rapid prototyping technology to assist in microcatheter shaping. The authors created a 3D solid aneurysm model based on digital imaging and communications in medicine (DICOM) data to guide microcatheter shaping, followed by the use of a hollow 3D aneurysm model to test the accuracy of the shaping. Subsequently, the pre-shaped microcatheter was used to perform coil embolization treatment. Ultimately, in their series of cases, all 10 patients achieved accurate microcatheter shaping. Toshihiro et al. (10) and Zeng et al. (41) expanded the number of cases using this shaping method. In their report, a total of 27 aneurysms and 31 aneurysms were treated with the assistance of 3D printing technology for microcatheter shaping, respectively, and the authors concluded that this tailor-made shaping method yielded satisfactory outcomes.

**Table 3:** Summary of published studies 3D model reconstruction and printing technology.

**Study**	**Number of aneurysms**	**Type of microcatheters**	**DICOM**	**Shaping method**	**Shaping reference**	***In vitro* validation**	***In vivo* performance**	**Immediate outcome**
Namba et al. (40)	10	Echelon-10	3D-DSA	Manually mandrel bent	Solid 3D vessel model	Hollow 3D aneurysm model	Appropriate position 10 (100%)	Complete occlusion 5 (50.0%)
		Excelsior 1018					Stability 9 (90.0%)	Neck remnant 3 (30.0%)
								Residual aneurysm 2 (20.0%)
Ishibashi et al. (10)	27	Excelsior SL-10	3D-DSA	Manually mandrel bent	Solid 3D vessel model	-	Appropriate position 20 (74.1%)	-
		Headway-17						
		Echelon-10						
		Excelsior 1018						
Xu et al. (42)	13	Headway-17	3D-DSA	Manually mandrel bent	3D-DSA	Solid 3D microcatheter model	Appropriate position 13 (100%)	-
							Stability 13 (100%)	
Quan et al. (43)	30	-	CTA	Manually mandrel bent	Profile 3D vessel model	Profile 3D vessel model	Appropriate position 30 (100%)	Complete occlusion 30 (100%)
							Stability 30 (100%)	
Xu et al. (8)	9	Excelsior SL-10	CTA	Intra-model placement with heating water	Hollow 3D aneurysm model	Hollow 3D aneurysm model	Appropriate position 8 (88.9%)	Complete occlusion 5 (55.6%)
							Stability 9 (100%)	Neck remnant 3 (33.3%)
								Residual aneurysm 1 (11.1%)
Nakajima et al. (44)	14	Headway 17	3D-DSA	Mandrel inserting into the hollow model	Hollow 3D aneurysm model	Hollow 3D aneurysm model	Appropriate position 13 (92.9%)	-
		Excelsior SL-10					Stability 13 (92.9%)	
Song et al. (11)	16	Headway-17	3D-DSA	Intra-model placement with heating water	Hollow 3D aneurysm model	Hollow 3D aneurysm model	Appropriate position 13 (81.3%)	Complete occlusion 14 (87.5%)
							Stability 15 (93.8%)	Residual aneurysm 2 (12.5%)
Zeng et al. (41)	31	Echelon 10	CTA	Manually mandrel bent	Hollow 3D aneurysm model	Hollow 3D aneurysm model	Appropriate position 22 (70.9%),	-
		Echelon 14					Stability 26 (83.9%)	

DICOM, digital imaging and communications in medicine; 3D-DSA, 3D rotational digital subtraction angiography; CTA, computed tomography angiography.

Xu et al. (8) adopted a more ingenious approach to utilize 3D printing technology in assisting microcatheter shaping. They created hollow and translucent 3D models that were immersed in water. Subsequently, the microcatheter was introduced into the target position in the models, and the water temperature was heated to 50°C for 5 min to achieve the desired shape of the microcatheter. Finally, the embolization procedures were successfully performed. In another study, Song et al. (11) applied the same method to treat 16 patients with aneurysms. Additionally, they made a small improvement to this method by creating perforators on the surface of the model to facilitate steam heating. In a similar manner, Nakajima et al. (44) inserted a mandrel into a 3D-printed hollow model to create an ideal shape to assist in shaping the microcatheter under the condition of heating with a hot air gun.

3D reconstruction modeling and printing technology provided operators with a relatively accurate and customized microcatheter shaping aid for specific patients, which theoretically facilitated the implementation of endovascular treatment procedure and improved its safety. However, this method still had certain limitations. Due to the process of obtaining DICOM data and forming the models, as well as the time required for model sterilization, it typically takes ~1 day. Therefore, for patients requiring emergency operations for ruptured aneurysms, the application of 3D printing technology posed difficulties.

### Computer simulation technology assisted shaping

In recent years, with the continuous development of computer technology, computer algorithm-based simulation techniques have gradually been applied in the field of cerebral endovascular therapy (45–47). Song et al. (48) introduced an intelligent microcatheter shaping method platform named UKnow, developed by Union Strong (Beijing) Technology Co. Ltd., based on a computer algorithm model, and validated it on the *in vitro* silicone models, which provided a novel method and system for assisting intracranial aneurysm embolization. In their algorithm model, the computer software performed a 3D reconstruction of the parent artery and aneurysm based on the acquired DICOM data, utilized “collision detection” and “centerline constraint” algorithms for path simulation and correction, as well as automatically generated the expected microcatheter shape and mandrel shape. Finally, the interventionalist applied the computer-generated solution to the physical microcatheter and mandrel for shaping operations.

**Figure 3:**
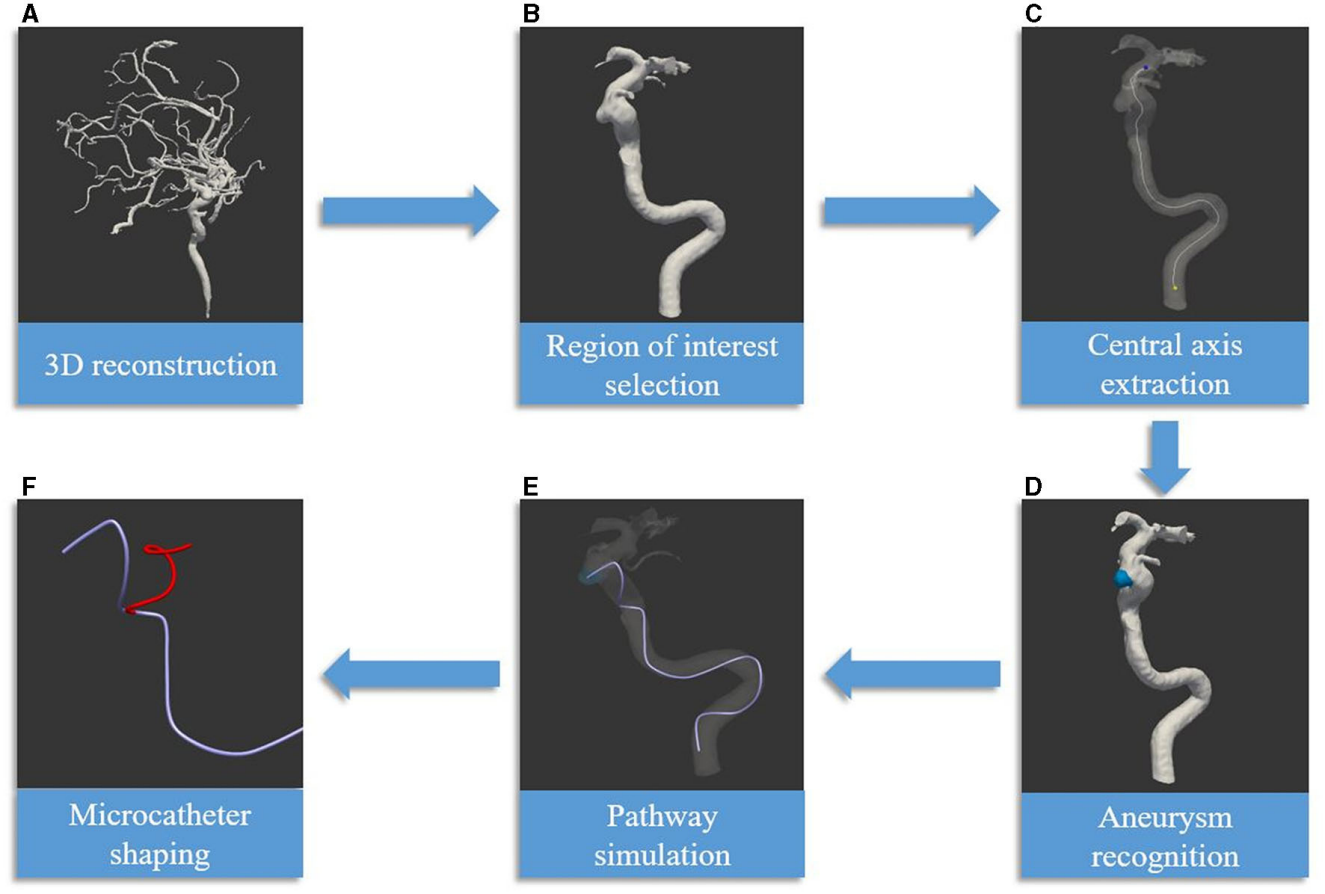
Delineates the process of employing artificial intelligence technology in the shaping of microcatheters [reprinted with permission from Liu et al. (6), © 2022 Elsevier Inc.] **(A)** The procedure begins with the generation of a three-dimensional vascular image, constructed using DICOM data obtained from CTA or DSA. **(B)** The subsequent phase involves the segmentation of arteries within the defined region of interest. **(C)** The centerline is then extracted, serving dual purposes: as the simulation path and as the central axis of the parent artery. **(D)** The process progresses with the detection of the aneurysm, along with the determination of the target for the microcatheter tip. **(E)** The artificial intelligence algorithm is then utilized to simulate the path of the microcatheter. **(F)** The final phase encompasses the creation of the mandrel and microcatheter shapes. This is based on the simulated path and the characteristics of various microcatheter types. The red line symbolizes the optimal mandrel shape, determined through software-calculated parameters and the elasticity features of the microcatheter, exemplified here by the Echelon microcatheter.

**Figure 4:**
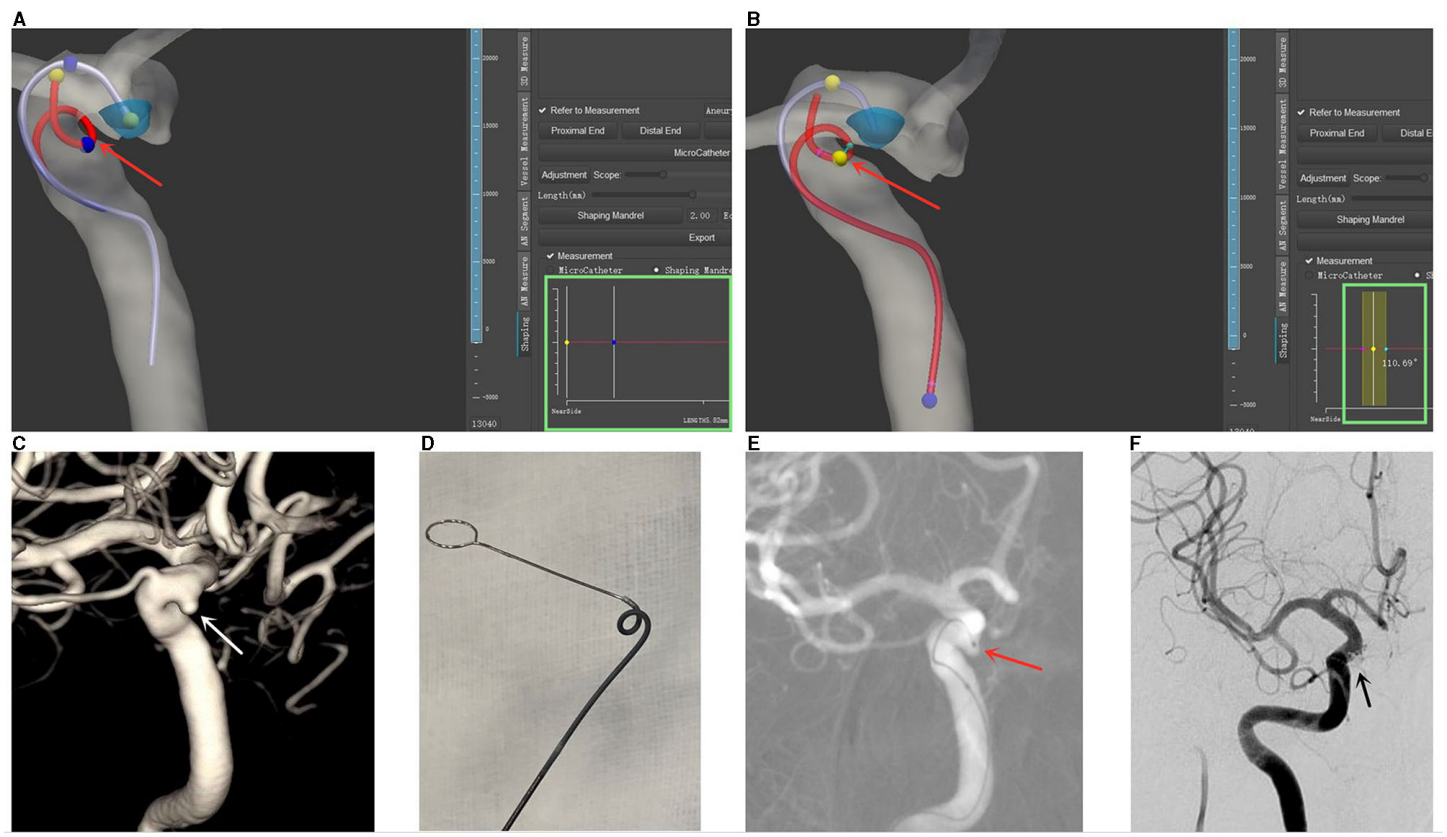
Illustrative case demonstrating computer-assisted microcatheter shaping [reprinted with permission from Liu et al. (6), © 2022 Elsevier Inc.]. **(A)** Three-dimensional (3D) reconstructions of the aneurysm and the parent artery were generated in the software. The planned trajectory and form of an Echelon microcatheter are denoted by a purple line, while the simulated shaping mandrel is represented by a red line. Adjustable markers in yellow and blue (highlighted by a red arrow) permit the calculation of the distance between them, which is displayed in the green box (measured distance: 5.82 mm). **(B)** Angular measurements are also enabled at each curve of the simulated shaping mandrel, as indicated by a red arrow. The calculated angle is displayed in the green box (measured angle: 110.69°). **(C)** The aneurysm is visualized through 3D-DSA, marked by a white arrow. **(D)** The microcatheter is shaped *in vitro*, adhering to the pre-calculated trajectory and form. **(E)** The shaped microcatheter is successfully utilized to access the aneurysm sac on the first attempt, and the tip positioning is deemed satisfactory, as indicated by a red arrow. **(F)** Successful embolization of the aneurysm is achieved, as denoted by a black arrow.

Based on this software and algorithm (illustrated in Figure 3) (6), we conducted a preliminary study on its clinical application. We utilized this microcatheter shaping technique to treat a total of 30 aneurysms in 24 patients. Ultimately, all 30 microcatheters accurately entered the aneurysmal sac, while 25 achieved satisfactory results in terms of position within the sac and intraoperative stability (illustrated in Figure 4) (6). In a recently published multicenter randomized controlled study on the clinical application of this software (49), 101 patients underwent treatment with computer-assisted microcatheter shaping technology (CAMS group), while another 100 patients underwent conventional manual microcatheter shaping methods (MMS group). The result indicated that the CAMS group showed significant superiority over the MMS group in terms of the success rate of the first attempt (96.0 vs. 66.0%, *P* < 0.001), success rate of microcatheter placement within 5 min (96.0 vs. 72.0%, *P* < 0.001), microcatheter stability (97.0 vs. 84.0%, *P* = 0.002), and excellent delivery performance without microwire guidance (45.5 vs. 24.0%, *P* < 0.001). At the same time, Wu et al. (50). reported on another computer simulation-assisted microcatheter shaping system, AneuShapeTM, which was developed by ArteryFlow Technology Corporation, Hangzhou, China. They applied this system to treat 55 aneurysms, achieved favorable results in terms of accessibility (45/55, 81.8%), positioning (47/55, 85.5%), and stability (46/55, 83.6%), and demonstrated a promising outcome. Furthermore, Gangqin Xu et al. (51) reported a method based on computational fluid dynamics for simulating cerebral blood flow streamline to assist microcatheter shaping, which was successfully applied to seven patients.

The emergence of computer algorithm-assisted solution has provided convenience for microcatheter shaping and decision-making during the neurointerventional procedure, particularly for insufficiently experienced interventionalists. It overcame the limitations of the conventional shaping method that heavily relies on the interventionalist's experience, resulting in less accurate shaping. Additionally, it offered a potential advantage in enhancing the safety of procedure. However, it should be noted that how to precisely translate computer-generated shaping schemes to actual mandrels and microcatheters on a 1:1 scale, rather than relying on observation and manual manipulation by the interventionalist, remains an issue that computer simulation technology-assisted shaping needs to confront.

### Shaping with steerable articulating tip

In recent years, the introduction of the steerable guidewire and microcatheter has provided a new option for interventional procedures (52, 53). Due to the characteristic of a manipulable bending tip, the steerable guidewire and microcatheter also present a new paradigm for neurointerventionalists in terms of microcatheter shaping and navigation technique. Recently, studies have reported the clinical applications of a microcatheter named Bendit in the field of neurointervention, especially for cases that are traditionally challenging to manage, such as those where intravascular navigation is difficult (54–56). The steerable microcatheter has demonstrated commendable controllability, impressively navigating through intricate vascular structures. Currently, while the application of steerable microcatheters in neurointervention is still in its preliminary phase with small sample sizes, it is believed that with continuous improvements in material and design, along with accumulating clinical experience, steerable microcatheters will offer more possibilities in neurointerventional techniques, ultimately benefiting patients.

## Conclusion

In recent years, with the development of neurointerventional theory, techniques, and materials, microcatheter shaping, as an important part of neurointerventional procedure, has undergone various advancements. The shaping methods have evolved from conventional manual shaping to incorporating composite bending, then to 3D model assistance, and now to the emergence of computer algorithm-assisted techniques. Researchers and interventionalists have been continuously exploring and improving these techniques. Accurate insertion and stable placement of microcatheters within aneurysmal sacs are crucial for the success of interventional embolization procedures. Therefore, achieving greater accuracy in microcatheter shaping is an ongoing goal for every interventionalist. In future, the combination of computer algorithm assistance and 3D model printing under sterile conditions in the operating room may represent a new direction and application for the microcatheter shaping method. We believe that the development of these methods and technologies will bring more benefits to interventionalists and patients.

## Author contributions

BF, XH, and CL: conception and design. CL and XW: analysis, interpretation of the data, article drafting, and review of the submitted version of manuscript. BF and SZ: article revision. BF, SZ, and CL: final approval of the version to be published. XH, LW, and KG: technical and material support. All authors contributed to the article and approved the submitted version.
